# Clinical characteristics and economic burden of Alzheimer’s disease inpatients in Hubei Province, China: a retrospective analysis of hospitalization costs and length of stay

**DOI:** 10.3389/fpubh.2025.1595904

**Published:** 2025-05-27

**Authors:** Siyi Chen, Chenguang Jia, Liangzi Zhang, Yin Shen, Da Zhou, Meng Li, Xinye Peng, Wen Liu

**Affiliations:** ^1^Department of Pharmacy, Zhongnan Hospital of Wuhan University, Wuhan University, Wuhan, China; ^2^Department of Neurosurgery, Affiliated Hospital of Hebei University, Baoding, China; ^3^Division of Personnel Services, Zhongnan Hospital of Wuhan University, Wuhan, China; ^4^Department of Integrative Ultrasound Medicine, Zhongnan Hospital of Wuhan University, Wuhan, China; ^5^Center for Health Information and Statistics of Hubei, Wuhan, China; ^6^Department of Finance, Zhongnan Hospital of Wuhan University, Wuhan, China; ^7^Department of Neurosurgery, Zhongnan Hospital of Wuhan University, Wuhan, China

**Keywords:** Alzheimer’s disease, hospitalization cost, clinical features, aging, insurance

## Abstract

**Background:**

The rapid aging of the population in China has led to a significant increase in the incidence of Alzheimer’s disease (AD). This escalating trend has resulted in a substantial economic burden, posing a formidable challenge to society.

**Methods:**

The study population comprised inpatients with AD in Hubei Province from January 2019 to December 2021. Comprehensive patient information was extracted from the provincial inpatient electronic system database. The data collected included age, gender, occupation, insurance type, method of hospital admission, diagnosis, length of stay (LOS), total medical expenses (TME), and discharge condition. Multiple linear regression analysis was employed to identify and analyze the factors influencing LOS and TME among AD patients.

**Results:**

The study encompassed a total of 22,301 AD patients. The mean age of the patients was 79.58 ± 10.12 years, with over 90% of the AD patients being 65 years or older. Male patients constituted 49.94% of the study population. The average LOS was determined to be 19.35 days. The mean TME per patient was calculated at US$2,593.38. A positive correlation was observed between medical expenses and patient age. Notably, the medical expenses for patients aged 85 years and above were 2.14 times higher than those for patients under 65 years. Of the total expenses, 57.04% were allocated to medication and service fees. Regarding comorbidities, infections, fractures, and cardiovascular diseases were identified as the top three cost drivers for AD inpatient hospitalization.

**Conclusion:**

Age and insurance type were identified as key determinants of both LOS and TME. To address these issues, strategies should be implemented to expand medical insurance coverage and enhance daily care for AD patients. Furthermore, it is crucial to prioritize the prevention of infections, fractures, and cardiovascular diseases among AD patients. The implementation of comprehensive health policies focusing on drug pricing, diagnostic procedures, and service costs is essential to mitigate the economic burden associated with AD.

## 1 Introduction

The incidence of Alzheimer’s disease (AD) has risen dramatically with the rapid progression of societal aging (1). It has been reported that over 35 million individuals globally are afflicted with dementia. This population is projected to escalate to 65 million by 2030 and 115 million by 2050 (2). In China, the prevalence of AD has accelerated due to the expanding older adult population, mirroring global epidemiological trends. Among individuals aged 60 and above, approximately 15.5% have been diagnosed with mild cognitive impairment (MCI) (3). Consequently, AD has emerged as one of the most formidable health challenges confronting China’s aging populace.

The burden imposed by neurological disorders has intensified significantly over the past decade. The Global Burden of Disease (GBD) study 2017 identified AD as one of the three most burdensome neurological conditions in the United States (4). In China, the societal and economic ramifications of AD are becoming increasingly pronounced. A national study conducted in 2015 estimated the annual medical expenditure for AD at US$167.74 billion. More alarmingly, this cost is projected to surge to US$1.8 trillion by 2050 (5). However, the diagnosis rate of AD in China remains sub-optimal (6), leading to a substantial underestimation of the economic burden associated with this disease.

AD imposes a significant economic burden on families due to its chronic progression and high prevalence of comorbidities, which exacerbate healthcare resource demands (5). Emerging evidence reveals that specific clinical variables, including advanced age, gender disparities, functional disability, and cognitive deterioration, act as key cost drivers by prolonging hospitalization and amplifying dependence on long-term care (7). Current cost-of-illness studies typically categorize AD-related expenditures into two domains (8). Direct costs encompass expenditures related to diagnosis, treatment, hospitalization, and follow-up care. Indirect costs, conversely, refer to disease-related resource depletion (9), specifically the reduced productivity of both the AD patient and their family members (2). While direct costs are more readily quantifiable, indirect costs present greater challenges in assessment. Nevertheless, there is a paucity of research examining the medical costs incurred by AD patients in China. Furthermore, the factors influencing the length of hospital stay and associated medical expenses remain inadequately elucidated.

This study aims to comprehensively evaluate the clinical features and medical expenses of AD inpatients in Hubei Province, China, from 2019 to 2021. The research will elucidate key factors influencing the duration of hospitalization and medical expenditures for AD patients. Additionally, the study will investigate potential reasons for AD-related hospitalizations and provide a detailed analysis of the medical costs associated with diagnosis, treatment, and medication during hospital stays.

## 2 Materials and methods

### 2.1 Data source

Hubei Province, a central region of China, boasts a permanent population of nearly 60 million residents. According to the China Alzheimer Report 2022 (10), approximately 600,000 patients with AD reside in Hubei Province. The rapid increase in the aging population and socioeconomic factors has led to a continuous rise in AD incidence rates. To enhance health service quality supervision and comprehensively evaluate medical costs, MRs have been implemented in Hubei Province since 2016. These electronic medical records (EMRs) encompass inpatient cost data from ‌all 929 public hospitals‌ across the province. The present study conducted a comprehensive analysis of AD patients from 2019 to 2021 utilizing the inpatient electronic system database in Hubei Province. Data extracted included age, gender, occupation, insurance type, hospitalization admission method, LOS, TME, diagnosis, primary reasons for hospitalization, and discharge condition. To safeguard patient privacy, crucial information such as names, home addresses, ID card numbers, social security card numbers, and contact details were anonymized. This study received approval from the Ethics Committee of Zhongnan Hospital, Wuhan University (Approval No. 2023065 K).

### 2.2 Patient information

This investigation focused on the clinical features and medical expenses of AD inpatients, with particular emphasis on LOS, reasons for hospitalization, and AD-related hospital costs. Cases diagnosed with AD prior to or newly diagnosed between 2019 and 2021 were extracted from the EMR system. The diagnostic criteria for AD were based on the standards proposed by the National Institute of Neurological and Communicative Disorders and Stroke–Alzheimer’s Disease and Related Disorders Association (NINCDS–ADRDA) in 1984 (11). Diagnoses were made by specialist physicians. Patients without an AD diagnosis were excluded from this study.

In this study, inpatient information was extracted, comprising two primary components: basic clinical characteristics and detailed disease information, along with associated medical expenses.

(1) Basic clinical characteristics were obtained from MRs, including patient demographics, age, gender, and occupational categories.

(2) Detailed disease information was extracted, encompassing primary diagnoses, reasons for hospitalization, comorbidity data, admission types, and discharge statuses.

(3) Medical expense data were collected, including payment methods, LOS, TME, and costs related to comorbidities. Gender-based differences in medical expenses were analyzed. Additionally, various age groups and comorbidity categories were examined to elucidate the relationship between medical expenses and AD. Regression analysis was employed to investigate factors correlating with LOS and medical expenses of AD patients. The independent variables explicitly included ‌age, gender, occupation, and insurance type‌. The covariates included in the regression models (age, gender, occupation, and insurance type) were selected based on their hypothesized or documented associations with healthcare utilization and costs in Alzheimer’s disease populations (8, 12). This comprehensive information facilitates the evaluation of inpatient health service utilization efficiency.

Payment methods for medical expenses are categorized into insurance-based and personal payments, contingent upon insurance coverage types. The Urban Employee Basic Medical Insurance (UEBMI) system, designed for urban employees and retirees, offers a relatively high reimbursement ratio and comprehensive medical coverage. In contrast, the Urban Resident Basic Medical Insurance (URBMI) system, established for urban residents without employer-provided insurance, primarily relies on personal payments with supplementary government subsidies to cover basic medical needs. Reimbursement for drugs or consumables under these insurance schemes is restricted to the scope defined by the China National Healthcare Security Administration. Commercial health insurance in China serves as a private, supplementary option to the basic medical security system. Compared to government-provided basic medical insurance, commercial health insurance presents a broader range of protection options and more flexible insurance products for individuals.

### 2.3 Statistical analysis

Data analysis was conducted using SPSS 21.0 software. Continuous variables were presented as mean ± standard deviation (SD). Variables following a normal distribution were subjected to T-test analysis. Multiple linear regression analysis was applied to explore factors correlating with LOS and medical expenses of AD patients. Statistical significance was established at *p* < 0.05.

## 3 Results

### 3.1 Clinical characteristics of AD patients

From January 2019 to December 2021, 22,301 patients with AD sought medical treatment in Hubei Province (Table 1). The average LOS was 19.35 days, with a mean total medical expense (TME) of US$2,593.38 per patient. In 2019, 6,719 AD patients were hospitalized, with an average LOS of 17.8 days and a mean TME of US$2,517.03. Despite a lower daily medical expense (US$141.41) compared to the national average (US$156.88), the extended hospital stay resulted in a higher TME (US$2,517.03) than the national average (US$1,427.62). In 2020, 6,283 AD patients were hospitalized, with an average LOS of 22.15 days and a mean TME of US$2,793.54. In 2021, 9,299 AD patients received hospital treatment, with an average LOS of 18.58 days and a mean TME of US$2,676.10 (Table 1).

**Table 1:** The inpatient expenses of Alzheimer’s disease in Hubei Province during 2019–2021.

Year	AD population	AD medical expenses in Hubei			National medical expenses		
		Average LOS (Day)	Average TME ($)	DME ($)	Average LOS (Day)	Average TME ($)	DME ($)
2019	6,719	17.80	2,517.03	141.41	9.1	1,427.62	156.88
2020	6,283	22.15	2,793.54	126.12	8.5	1,539.57	181.13
2021	9,299	18.58	2,676.10	144.03	9.2	1,705.45	185.38

AD, Alzheimer’s disease; LOS, length of stay; TME, total medical expenses; DME, daily medical expenses in hospital.

Temporal trends in clinical characteristics (2019–2021) is show in the fellow: 1. hospitalized AD population: a decline of 6.5% (6,719 to 6,283 patients) occurred from 2019 to 2020, followed by a sharp increase of 48.0% (6,283 to 9,299 patients) in 2021. This rebound may reflect delayed hospital visits during the COVID-19 pandemic in 2020 and subsequent catch-up care in 2021; 2. average LOS: LOS increased by 24.4% (17.8 to 22.15 days) in 2020 compared to 2019, likely due to pandemic-related complications or stricter discharge protocols. By 2021, LOS decreased by 16.1% (22.15 to 18.58 days), approaching pre-pandemic levels; 3.TME: TME rose by 11.0% (US 2517.03 to US2,793.54) in 2020 despite reduced daily medical expenses (DME: US 141.41 to US 126.12), driven by prolonged LOS. In 2021, TME slightly decreased by 4.2% (US 2793.54 to US 2676.10), aligning with shorter LOS.

Among the 22,301 AD patients, the mean age was 79.58 ± 10.12 years, with over 90% of patients aged 65 years or older (Table 2). The gender distribution was nearly equal, with 11,137 (49.94%) male patients. Regarding occupation, the majority of AD patients were retired (57.35%), while 2,401 (10.74%) were unemployed. In terms of medical insurance, 3,974 (17.82%) had urban resident basic medical insurance, and 12,210 (54.75%) had employee basic medical insurance, primarily due to their retired status. Notably, 3,430 (15.38%) patients lacked medical insurance and opted for self-payment for hospitalization. The majority of AD inpatients (77.07%) were admitted through outpatient services, while 3,102 (13.91%) were admitted through emergency services. Upon discharge, 20,290 (90.98%) patients experienced symptom relief following hospitalization (Table 2).

**Table 2:** The basic characteristics of hospitalized AD patients in Hubei Province during 2019–2021.

Characteristic	Year		
	2019	2020	2021
Age	79.42 ± 9.97	79.66 ± 9.93	79.65 ± 10.35
<65 years old	687 (10.22%)	594 (9.45%)	965 (10.38%)
65-85 years old	3,975 (59.16%)	3,473 (55.28%)	5,302 (57.02%)
>85 years old	2,057 (30.61%)	2,216 (35.27%)	3,032 (32.60%)
Gender			
Male	3,433 (51.24%)	3,106 (49.43%)	4,598 (49.45%)
Female	3,286 (48.76%)	3,177 (50.57%)	4,701 (50.55%)
Occupation			
Employed	258 (3.84%)	186 (2.96%)	221 (2.38%)
Unemployed	680 (10.12%)	701 (11.15%)	1,020 (10.97%)
Farmers	482 (7.17%)	464 (7.39%)	614 (6.60%)
Retired	3,851 (57.32%)	3,693 (58.78%)	5,245 (56.40%)
Others	1,445 (21.55%)	1,239 (19.72%)	2,199 (23.65%)
Insurance type			
Urban resident basic medical insurance	1,181 (17.58%)	1,101 (17.52%)	1,692 (18.20%)
Employee basic medical insurance	3,612 (53.76%)	3,559 (56.64%)	5,039 (54.18%)
Business insurance or other insurance	782 (11.64%)	703 (11.19%)	1,202 (12.93%)
Self-pay	1,144 (17.02%)	920 (14.65%)	1,366 (14.69%)
Discharge condition			
Healed or improved	6,055 (90.12%)	5,666 (90.18%)	8,569 (92.15%)
Unhealed	18 (0.27%)	36 (0.57%)	40 (0.43%)
Other	646 (9.61%)	581 (9.25%)	690 (7.42%)

### 3.2 Medical expenses of AD patients

**Figure 1:**
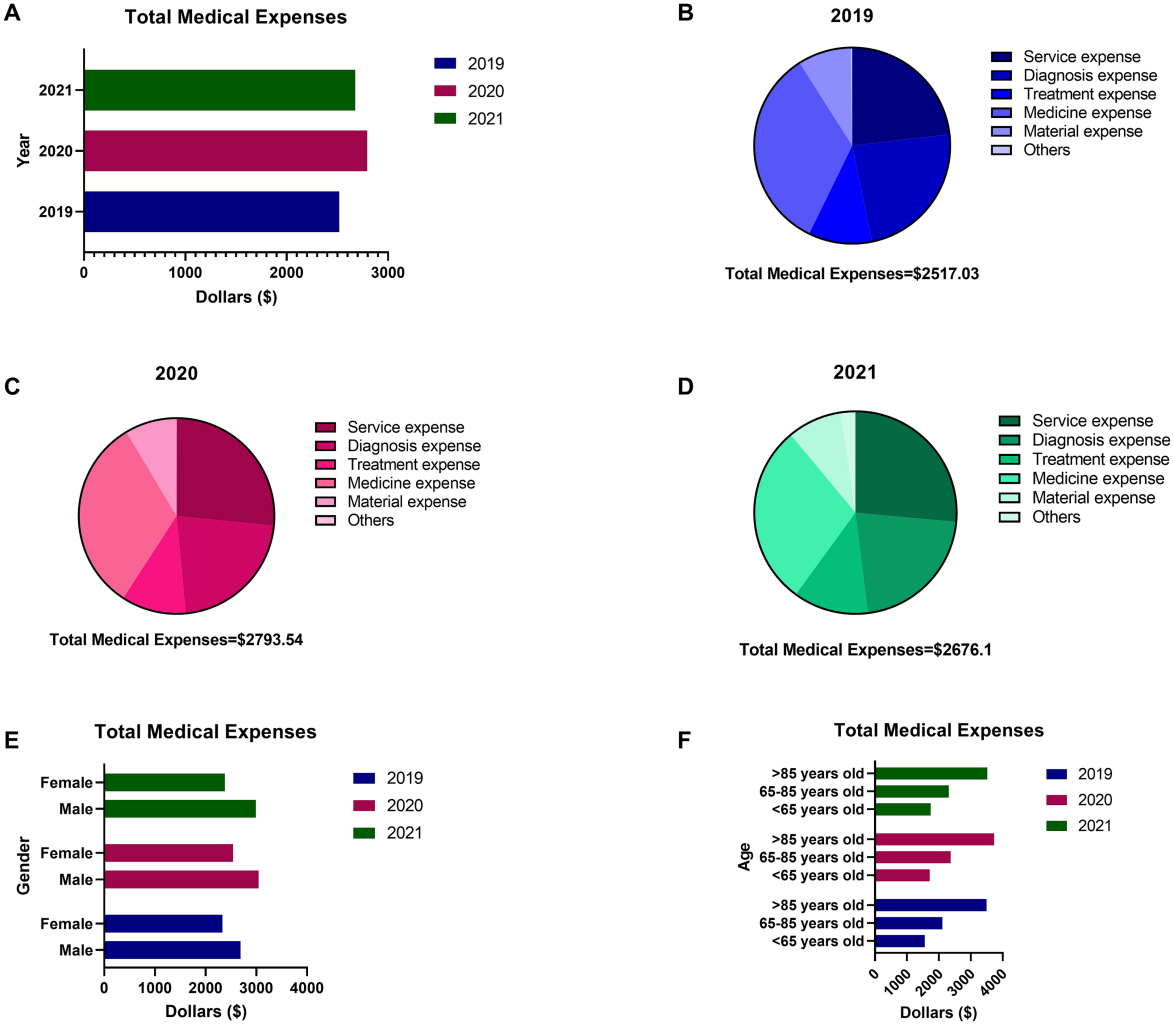
The total medical expenses (TME) of inpatients with AD during 2019–2021 in Hubei Province. **(A)** The average TME of inpatients with AD in 2019, 2020 and 2021. **(B)** The details of medical expense of 2019. **(C)** The details of medical expense of 2020. **(D)** The details of medical expense of 2021. **(E)** The difference of TME between males and females. **(F)** The difference of TME among different age groups.

The medical expenses associated with AD represent a significant economic burden for patients’ families. Data from 2019 to 2021 reveal that the average TME exceeded US$2,500 (Figure 1A). Of the total expenses, 57.04% were allocated to medicine and service fees, with the remainder spent on diagnosis, treatment, and materials (Figures 1B–D). An analysis of gender and age factors on average medical expenses revealed that male patients consistently incurred higher medical expenses than female patients from 2019 to 2021 (Figure 1E). Additionally, medical expenses demonstrated a positive correlation with patient age (Figure 1F). Notably, the medical expenses for patients aged 85 years and older were 2.14 times higher than those for patients under 65 years of age.

### 3.3 Correlation factors of length of stay and medical expenses in AD patients

Multiple regression analysis was applied to the database to investigate potential influencing factors associated with LOS (Table 3) and TME (Table 4) in AD patients. For LOS, age (*β* = 0.08, *p* < 0.001) and unemployment status (*β* = 1.96, *p* = 0.03) were positively correlated with prolonged hospitalization. Conversely, patients covered by employee basic medical insurance (*β* = −1.28, *p* = 0.001), commercial insurance (*β* = −1.52, *p* = 0.001), or self-pay status (*β* = −1.02, *p* = 0.011) had significantly shorter LOS compared to those with urban resident basic medical insurance. For TME, age exhibited the strongest positive association (*β* = 62.53, *p* < 0.001), with each additional year increasing costs by approximately US$ 62.53. Notably, commercial insurance was a significant driver of higher expenses (*β* = 530.08, *p* < 0.001) compared to urban resident basic medical insurance.

**Table 3:** The regression analysis of correlation factors on LOS of AD patients.

Variable	*B*	S.E.	*β*	*t*	*P*	95% CI lower	95% CI upper
(Constant)	12.38	1.45		8.53	0.000	9.54	15.23
Age	0.08	0.01	0.04	6.06	0.000	0.06	0.11
Gender							
Female	−0.28	0.27	−0.01	−1.02	0.310	−0.80	0.25
Male							
Occupation							
Unemployed	1.96	0.09	0.03	2.18	0.030	0.19	3.72
Farmers	−0.85	0.96	−0.01	−0.89	0.374	−2.72	1.02
Retired	1.48	0.82	0.04	1.81	0.070	−0.13	3.09
Others	−0.32	0.86	−0.01	−0.38	0.710	−2.00	1.36
Employed							
Insurance type							
Employee basic medical insurance	−1.28	0.40	−0.02	−3.19	0.001	−2.06	−0.49
Business insurance or other insurance	−1.52	0.45	−0.02	−3.41	0.001	−2.39	−0.65
Self-pay	−1.02	0.40	−0.02	−2.55	0.011	−1.80	−0.24
Urban resident basic medical insurance							

LOS, length of stay.

**Table 4:** The regression analysis of correlation factors on TME of AD patients.

Variable	*B*	S.E.	*β*	*t*	*P*	95% CI lower	95% CI upper
(Constant)	−2378.91	256.10		−9.29	0.000	−2880.883	−1876.94
Age	62.53	2.42	0.17	25.83	0.000	57.78	67.27
Gender							
Female	−60.40	47.42	−0.01	−1.27	0.200	−153.34	32.54
Male							
Occupation							
Unemployed	72.67	158.78	0.01	0.46	0.647	−238.55	383.88
Farmers	−166.27	168.59	−0.01	−0.99	0.324	−496.72	164.18
Retired	193.39	14,460	0.03	1.34	0.181	−90.04	476.81
Others	−62.84	150.87	−0.01	−0.42	0.677	−358.55	232.88
Employed							
Insurance type							
Employee basic medical insurance	−57.15	70.71	−0.01	−0.81	0.419	−195.75	81.45
Business insurance or other insurance	530.08	78.51	0.05	6.75	0.000	376.19	683.96
Self-pay	14.35	70.54	0.00	0.20	0.839	−123.90	152.61
Urban resident basic medical insurance							

TME, total medical expenses.

### 3.4 Hospitalization reasons for AD patients and associated medical expenses

**Figure 2:**
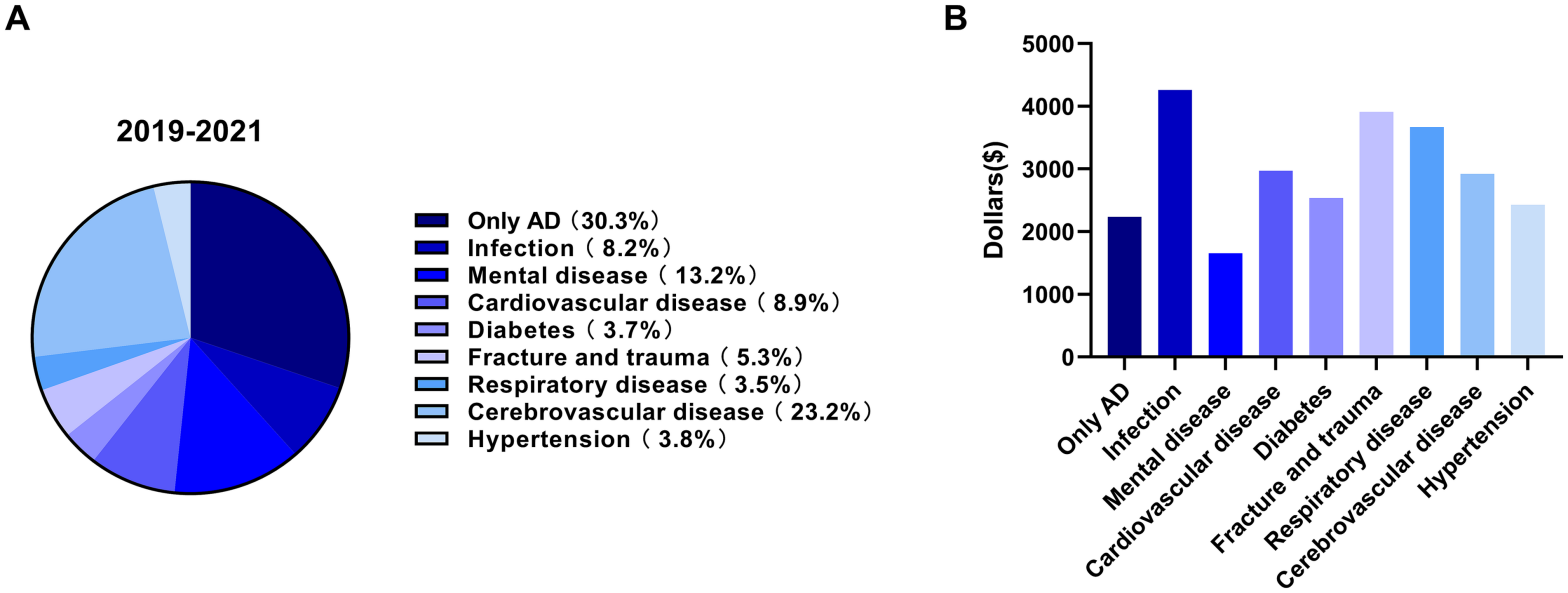
The analysis of reasons for hospitalization of AD patients and related medical cost of each disease. **(A)** The major reasons for hospitalization of AD patients from 2019 to 2021. **(B)** The medical expenses of each AD associated disease from 2019 to 2021.

Among the 22,301 patients analyzed, approximately 90% were aged 65 years or older. In addition to AD itself, cerebrovascular disease (23.2%), mental disorders (13.2%), and cardiovascular disease (8.9%) were identified as the three most common reasons for hospitalization (Figure 2A). Infections (8.2%), diabetes (3.7%), respiratory diseases (3.5%), hypertension (3.8%), and bone fractures (5.3$) were also frequently observed comorbidities that prompted AD patients to seek medical treatment. Regarding the medical expenses associated with comorbidities, infections, bone fractures, and cardiovascular diseases incurred higher costs compared to other conditions (Figure 2B). These findings suggest that implementing preventive measures against infections, fractures, and cardiovascular diseases could potentially serve as an effective strategy to reduce the overall medical expenses incurred by AD patients.

## 4 Discussion

The prevalence of AD among individuals aged 65 years and above is projected to reach 6.7% by 2030, with the total AD population estimated to surge to 23.2 million (12). This substantial increase in AD cases is anticipated to impose a significant economic burden on families and society. Between 1990 and 2010, national medical expenditures for AD or dementia in China escalated from US$ 0.9 billion to US$ 47.2 billion, representing a more than 50-fold increase. Notably, annual costs are projected to reach US$ 114.2 billion by 2030, posing a formidable challenge to China’s GDP (12). Hubei province has been reported to rank tenth in AD prevalence among various provinces in China, with a rate of 987.3 per 100,000 population (10). The present study analyzed data from 22,301 AD inpatients from 2019 to 2021, revealing an average age of 79.58 ± 10.12 years. The average LOS ranged from 17.8 to 22.15 days, while the average medical cost varied between US$ 2,517.03 and US$ 2,793.54. Both LOS and TME were observed to be higher than the national average (13, 14), underscoring the importance of investigating factors influencing these parameters in AD patients.

Previous research (15) has identified various risk factors associated with the development of neurological disorders, including birth weight, metabolic dysfunctions, and hypertension (16, 17). However, the specific risk factors affecting LOS and TME for AD patients in China remain poorly understood. To address this knowledge gap, multiple regression analysis was conducted to identify potential correlating factors. This study highlights the dual role of age and insurance type in shaping LOS and TME for AD patients. Older patients, with their complex comorbidities and care needs, incurred longer hospital stays and higher expenses. China’s recent healthcare policy reforms have significantly impacted AD-related costs. However, this study found that medication and service fees still accounted for 57.04% of total hospitalization costs, indicating limited coverage of high-cost innovative drugs and non-essential services under current policies. Based on these, it is imperative to implement strategies that expand medical insurance coverage within this age group (18). In addition, the main hospitalization triggers identified in this study, such as infections and fractures, and strengthening the capacity of primary care, including pneumonia vaccination, bone density testing, and fall prevention assessments, also help control medical costs (18).

This study found that male AD patients incurred significantly higher medical expenses than females, potentially linked to their greater cardiovascular disease burden (19). However, cultural factors, such as families’ prioritization of male health, may also indirectly influence healthcare decision-making, warranting further research. Commercial insurance was a significant driver of higher expenses. This disparity may reflect broader diagnostic coverage, access to advanced therapies, or utilization of private hospital services under commercial insurance plans, which often include fewer restrictions on high-cost treatments. Similar patterns have been observed in other studies, where commercial insurance is linked to increased healthcare expenditures due to reduced financial barriers to specialized care and advanced interventions. By contrast, public insurance schemes like UEBMI and URBMI, which adhere to standardized reimbursement protocols, may limit access to costly procedures, thereby curbing expenses. This study also found that unemployment status was significantly associated with prolonged hospital stay in AD patients (*β* = 1.96, *p* = 0.03). This result may reflect the systemic challenges faced by unemployed patients, such as insufficient medical payment capacity due to economic pressure, lack of home care resources after discharge, or weak social support networks.

Xu et al. reported that 1.09% of global GDP was attributed to AD-related costs (13). However, in China, AD costs accounted for 1.47% of GDP (5). In the present study, service fees, diagnostic fees, and medication expenses were identified as the top three components of economic burden for AD patients. A similar consumption trend and distribution have been observed in other diseases, such as Hepatitis B Virus (20). Consequently, the high amounts allocated to service fees, diagnostic fees, and medication expenses may be attributed to China’s healthcare infrastructure and policies. To mitigate medical costs, multiple healthcare policy reforms have been implemented in China (21, 22). The drug price zero-markup policy, which prohibits medical institutions from selling drugs to patients at prices higher than the purchase cost, was initially implemented in 2012, resulting in a 20.4% decrease in medication expenses. Recent reports indicate that this policy has not negatively impacted mortality rates or hospital readmissions. Nevertheless, AD patients continue to incur substantial expenses for medication, diagnostics, and services. To investigate potential cost drivers, an analysis of comorbidities among AD patients during hospitalization was conducted (15). The results revealed that cerebrovascular diseases, mental disorders, and cardiovascular diseases were the primary reasons for hospitalization, aside from AD itself. However, in terms of medical expenses, infections, fractures, and cardiovascular diseases were identified as the top three cost drivers for AD patients. Therefore, to reduce medical expenses, it is crucial to prevent the occurrence of infections, fractures, and strokes. Based on the study findings, specific interventions to prevent infections and fractures among AD patients can include: 1.provide free or low-cost influenza and pneumonia vaccinations for older adult AD patients; 2. implement government-funded home modification initiatives; 3. adopt integrated community care system, where multidisciplinary teams (nurses, physiotherapists, social workers) conduct regular home visits to monitor AD patients, manage comorbidities, and provide preventive care. These interventions could reduce complications, lower hospitalization costs, and alleviate the socioeconomic burden of AD in China.

The high dependence of the Chinese medical system on drugs and the shortcomings of outpatient services reflect the systematic differences in the management models of chronic diseases. Drawing on the experiences of Western outpatient priority, payment reform and community care, China needs to shift from “hospital-centered” to “patient-centered throughout the entire health cycle” in order to achieve the dual goals of medical cost control and service quality improvement against the backdrop of an increasingly aging population (23).

‌It is important to note that the study period overlapped with the COVID-19 pandemic, which may have introduced unique disruptions to healthcare utilization and costs, particularly in 2020. The pandemic likely influenced hospitalization patterns.‌ During 2020, lockdowns and restrictions on non-urgent care may have led to a reduction in hospitalizations for mild AD symptoms or routine follow-ups, resulting in a higher proportion of severe cases being admitted. This selection bias could partially explain the observed increase in average LOS and complication rates during 2020 (Table 1). In addition, patients avoiding hospitals due to fear of infection might have delayed care, leading to worsened conditions upon admission.

Several limitations should be acknowledged in the present study. First, the retrospective cross-sectional design precludes causal inferences between variables. Second, The database utilized contains information solely from inpatient AD cases in 939 public hospitals in Hubei Province during 2019–2021. Regional disparities in healthcare access within Hubei Province may introduce sampling bias, potentially affecting the generalizability of findings. For instance, resource-abundant regions might disproportionately attract patients, whereas under-resourced areas could face barriers to care access. Consequently, extrapolation of these results to other regions should be approached with caution. Third, the absence of key clinical and socioeconomic variables such as disease severity, cognitive status, and SES, which may confound the associations observed. Fourth, only direct hospital costs were evaluated, while indirect costs incurred by patients and their family members remained unclear. Estimating the productivity loss of AD patients and their family members is typically challenging, however, this factor represents a significant component of the social burden associated with AD. For instance, caregivers often face reduced working hours, early retirement, or even job abandonment to provide long-term care, resulting in substantial income loss and diminished labor market participation. The exclusion of such costs may lead to a systematic underestimation of AD’s socioeconomic impact, potentially skewing policymakers’ prioritization of resource allocation. Future studies should incorporate mixed-methods approaches, combining quantitative assessments of lost wages and qualitative analyses of caregiver strain, to comprehensively quantify these hidden burdens. Additionally, economic evaluations of AD interventions must consider indirect cost savings—such as reduced caregiving time through community-based support programs—to better inform cost-effectiveness analyses and health policy design.

## 5 Conclusion

This comprehensive analysis of 22,301 AD inpatients in Hubei Province from 2019 to 2021 has revealed significant insights. Age and insurance type have been identified as the primary factors influencing LOS and TME. Based on the observed associations, potential policy implications may include: expanding insurance coverage, prioritizing preventive care for infections, fractures, and cardiovascular diseases, evaluating targeted reforms in drug pricing and diagnostic service fees. These suggestions align with the study’s findings but require further validation through longitudinal analyses and cost-effectiveness evaluations. Policymakers should consider these associations while designing interventions to alleviate the socioeconomic burden of AD.

## Data Availability

The raw data supporting the conclusions of this article will be made available by the authors, without undue reservation.
